# The modified cross-suture technique for unilateral pulled-out anchor during all-inside meniscal repair

**DOI:** 10.1186/s12891-020-03502-z

**Published:** 2020-07-22

**Authors:** Jianlong Ni, Zhibin Shi, Lihong Fan, Ning Guo, Haoyu Wang, Xiaoqian Dang, Dichen Li

**Affiliations:** 1 First Department of Orthopaedics, The Second Affiliated Hospital of Xi’an Jiaotong University, No. 157 Xiwu Road, Xi’an, 710004 Shaanxi China; 2 Department of Anesthesiology and Operation, The Second Affiliated Hospital of Xi’an Jiaotong University, Xi’an, China; 3 State Key Laboratory for Manufacturing Systems Engineering, Xi’an Jiaotong University, Xi’an, China

**Keywords:** Meniscus injury, Arthroscopy, All-inside repair, Cross-suture

## Abstract

**Background:**

Meniscal repair has received increasing attention, but for inexperienced doctors, unilateral suture anchor pulling out may occur during all-inside meniscal repair, and the treatment outcome may be affected. When the errors happened intraoperatively, how to minimize the loss under guaranteeing of treatment effectiveness is a topic worth studying.

**Purpose:**

To explore the practicability and effectiveness of the modified cross-suture method for arthroscopic remediation of unilateral suture anchor pulling out of an all-inside meniscal repair system.

**Methods:**

From May 2014 to May 2017, 28 patients diagnosed with injuries of the meniscus and anterior cruciate ligaments (ACL) from the First Department of Orthopaedics of the Second Affiliated Hospital of Xi’an Jiaotong University were enrolled in the study as the observation group, including 18 males and 10 females with an average age of 25.5 ± 2.3 years (range 18–42 years). All patients underwent ACL reconstruction concurrently. All meniscus injuries were repaired with an all-inside meniscal repair technique, and 1–3 needles of unilateral suture anchor pulling out occurred intraoperatively. The modified cross-suture method was used to remedy the error of anchor pulling out and to eventually complete an effective repair. Another 30 patients who underwent ACL reconstruction and all-inside meniscal concurrently without unilateral suture anchor pulling out, including 20 males and 10 females with an average age of 26.3 ± 1.9 years (range 19–45 years), were enrolled as the control group. During postoperative follow-up, range of motion, Lachman test and pivot shift test were performed during the physical examination. The clinical healing of the meniscus was evaluated according to the Barrett standard. The meniscus healing status was also confirmed with magnetic resonance imaging (MRI). The function of the knee joint was evaluated according to the IKDC, Lysholm and Tegner scores.

**Results:**

Twenty-five patients in the observation group and 28 patients in the control group completed the follow-up, with an average follow-up of 18.4 ± 5.2 months. All operations were performed by the same surgeon. At the follow-up 1 year after the operation, the average knee ROM of the two groups was 125.2 ± 4.3 degrees and 124.7 ± 3.8 degrees, the clinical healing rate of the meniscus of the two groups was 92.0% (23/25) and 92.9% (26/28), the MRI healing rate of the menniscus of the two groups was 72.0% (18/25) and 71.4% (20/28), and the IKDC, Lysholm and Tegner scores of the two groups were 90.52 ± 2.8, 89.17 ± 3.1, and 6.81 ± 1.7 and 91.42 ± 1.9, 90.32 ± 3.4, and 7.02 ± 1.4, respectively. The differences were not statistically significant (*P* > 0.05).

**Conclusions:**

The modified cross-suture method is practicable and effective for arthroscopic remediation of unilateral suture anchor pulling out in an all-inside meniscal repair system.

## Background

Meniscus injury is a common and frequent sports injury, and its treatment should maintain the integrity of the meniscal structure and function as much as possible; therefore, meniscal repair has received increasing attention [1–4]. With the development of an arthroscopic technique and the popularity of the concept of preserving meniscal function, a growing number of doctors have begun to perform arthroscopic meniscal suture repair operations [5–7]; however, as a result of inadequate surgical experience, inadequate operation visibility and unskilled operation assistants, various technical errors may occur when meniscal suture repair is performed, particularly during all-inside meniscal suture repair [8–10]. For instance, errors may include using an improper suture pattern, iatrogenic meniscal or chondral injury, improper tensioning of the suture and pulling out of the suture anchor [11]. Once unilateral suture anchor for the all-inside meniscal repair system is pulled out, the suture will fail, and the treatment will be affected [11–13]. Therefore, we explored a modified cross-suture method as a remedy when unilateral suture anchor pulls out of the meniscal repair system during all-inside meniscal suture repair. The purpose of this study was to explore the practicability and effectiveness of the modified cross-suture method for arthroscopic remediation of unilateral suture anchor pulling out of an all-inside meniscal repair system. We hypothesized that the modified cross-suture method would be practicable and effective for remedying unilateral suture anchor pulling out during all-inside meniscal repair because this method provides the dual advantage of vertical sutures and horizontal sutures that can promote meniscus healing.

## Methods

### General information

From May 2014 to May 2017, the clinical data for patients with meniscus injury who were treated in the First Department of Orthopaedics of the Second Affiliated Hospital of Xi’an Jiaotong University were analysed retrospectively. The inclusion criteria for the observation group were as follows: unilateral suture anchor was pulled out during all-inside meniscal repair at the time of an ACL reconstructions; full-thickness vertical longitudinal tear of the meniscus; the tear was in the red zone or red-white zone of the body or posterior angle of the meniscus, such that distance from the tear to the meniscal synovial margin was generally less than 6 mm; no previous history of joint surgery was present; arthroscopic cartilage damage was an Outerbridge II degree and below; and minimum follow-up period was greater than 12 months. The inclusion criteria for the control group were the same as those for the observation group except for the criterion that unilateral suture anchor pulling out during all-inside meniscal repair. The patients of isolated meniscal repair were also excluded to avoid the poor meniscus healing.

A total of 28 patients were enrolled as the observation group, and 30 patients were enrolled as the control group in this study. All cases involved a combination of a meniscal tear and ACL rupture which was confirmed with magnetic resonance imaging (MRI) and physical examination. All patients underwent all-inside meniscal suture repair and ACL anatomic single-bundle reconstruction concurrently. The observation group had an average of 1.3 ± 0.2 needles (range 1–3 needles) of unilateral suture anchor pulling out during all-inside meniscal suture repair intraoperatively. The modified cross-suture method was used to remedy the unilateral suture anchor pulling out and to eventually complete an effective repair. The OMNISPAN™ meniscus repair system was used for all cases (DePuy Mitek, Inc., USA).

### Operative method

The ACL rupture and meniscal injury type were confirmed after anterolateral and anteromedial portal establishment. The location and length of the meniscal tear were recorded. ACL anatomic single-bundle reconstruction was performed with autologous semitendinosus and gracilis tendons. Meniscal suturing was performed when the ACL reconstruction was complete, but the tibial interference screw was not screwed down. The torn meniscal edge was freshened with a meniscal file, and the meniscal suture repair was finished with the OMNISPAN™ meniscus repair system.

**Fig. 1:**
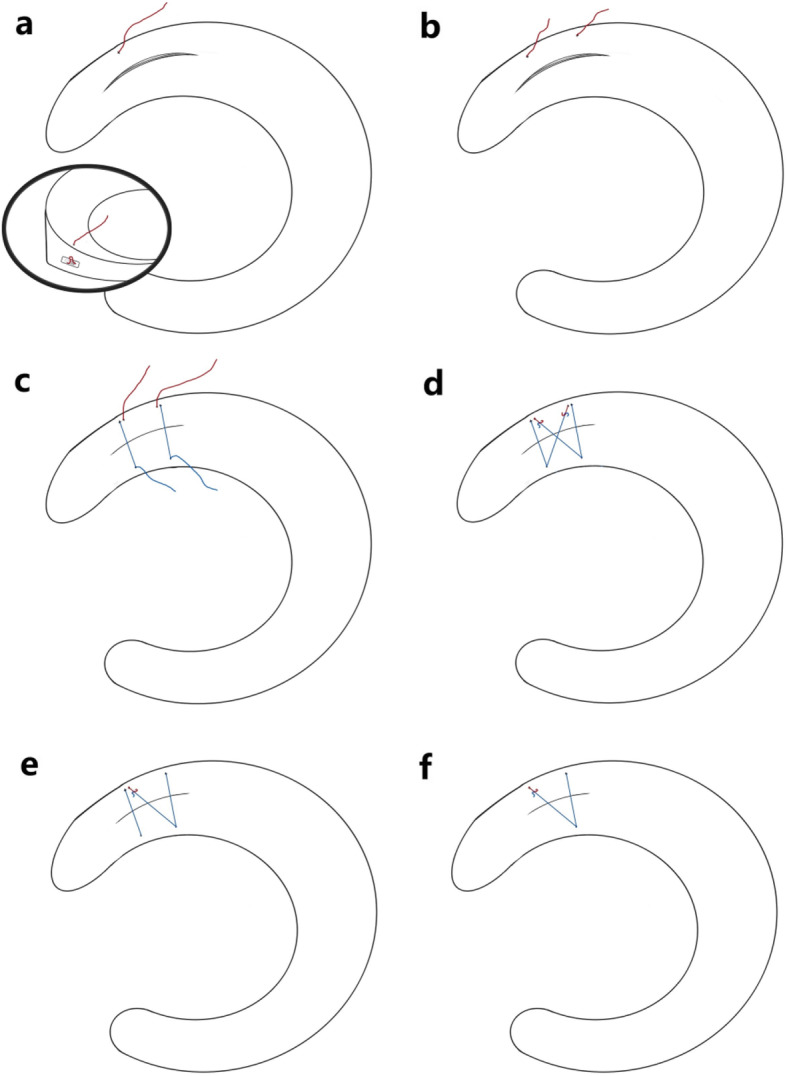
Schematic diagram of the “modified cross-suture”: **a**: Vertical longitudinal tear at the posterior angle of the medial meniscus. The suture (red line) in the diagram represents the free end of the suture of the all-inside meniscal repair system after unilateral anchor pulling out. **b**: The two sutures (red line) in the diagram represent the two free ends of the suture of the all-inside meniscal repair system after unilateral anchor pulling out. **c**: The two all-inside meniscal repair systems were reinstalled into the torn meniscus. The free suture leg (blue line) was pulled until the suture was tight against the meniscus surface and the meniscal tear was closed. The two free ends (blue line) were left at the opposite side of the standby suture (red line), as shown in Fig b. **d**: The two sutures (red line) of the unilateral anchor pulling out and the other two sutures (blue line) of normal state were cross-knotted to reinforce the meniscal tear. **e**: The cross-suture was finished if there was only one suture of unilateral anchor pulling out. **f**: If the length of meniscal tear was less than 10 mm, the cross-suture was finished more easily if there was only one suture of unilateral anchor pulling out

**Fig. 2:**
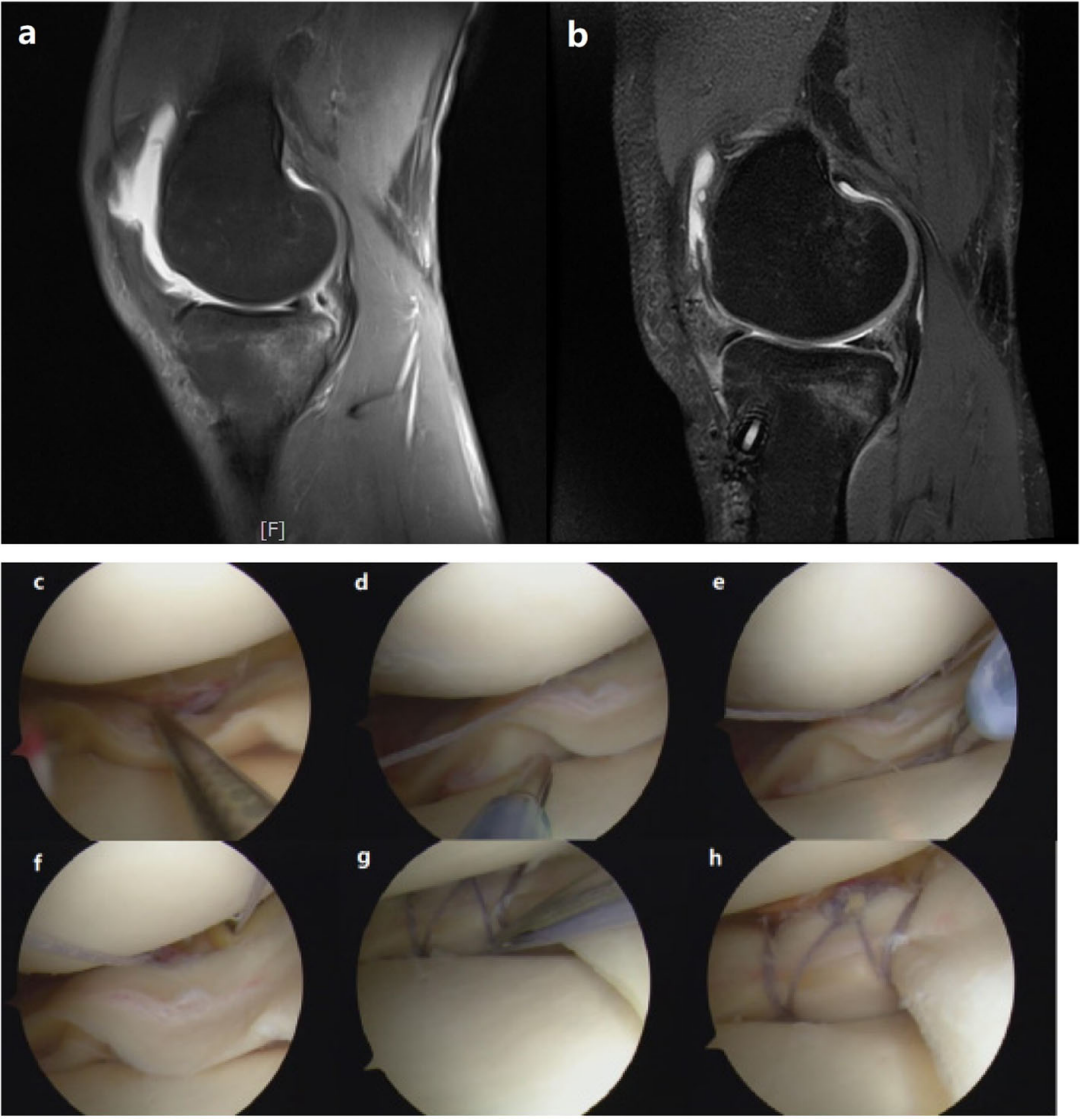
Typical case. The male patient was 33 years old and had an ACL rupture as well as a medial meniscal tear (**a**, **c**) in the right knee. The meniscus tear length was 2 cm. Two unilateral anchors of the all-inside meniscal repair system were pulled out intraoperatively (**d**, **e**, **f**). The modified cross-suture method (**g**) was used to remedy the error of unilateral suture anchor pulling out and to eventually complete an effective repair (**h**). According to the postoperative MRI 1 year postoperatively, meniscus healing was good (**b**)

If unilateral anchor pulling out occurred, the suture knot was pushed to the place of implantation of the other anchor at the synovial edge, and the free end of the suture was left for standby (Fig. 1a). The new OMNISPAN™ device was implanted and the suture knot was placed at the opposite side of the first device (Fig. 1c). The two free sutures were pulled out through the same portal and knotted with the shoulder arthroscopic knotting technique. The “cross-suture” type fixation was finished after the knot was tightened (Fig. 1e, f). If there were two anchor implants pulling out at the same time (Fig. 1b), a “double cross-suture” type fixation was used (Fig. 1d, Fig. 2).

### Postoperative management

The procedure was performed on an outpatient basis. Oral NSAIDs were used for 1 week. Normal full weight-bearing activities were allowed while wearing a knee extension brace for 6 weeks. Active and passive flexion was encouraged but limited to 90°. Walking and jogging were permitted at 3 months, and resumption of all sports activities and deep squatting were permitted 6 months after the operation.

### Postoperative follow-up and evaluation

The outpatient follow-up was conducted conventionally by the appointed surgeon. During the follow-up, a physical examination was performed to confirm knee mobility, including the ROM, Lachman test and pivot shift test. Knee function was evaluated by the International Knee Documentation Committee (IKDC), Lysholm and Tegner scoring systems. Meniscus healing was evaluated according to the Barrett standard [14]. If there was no joint swelling, joint space tenderness, or joint locking, and if the McMurray sign was negative, meniscal clinical healing was considered to be achieved; however, if one of these indicators was positive, the clinical healing requirements were considered to be unmet. The meniscus healing status was also confirmed with MRI 1 year postoperatively [15, 16]. On MRI, the meniscus was considered unhealed if Grade 3 signals on T2 sequences were seen. No patients underwent second-look arthroscopy in this study.

SPSS 20.0 statistical software (SPSS Inc., Chicago, IL, USA) was used for statistical analysis. The measurement data were expressed as the mean ± standard deviation (SD) or percentage of subjects. T-tests (if the variances are equal) or Mann-Whitney U-tests (if the variances are not equal) were used to evaluate the differences in the values of the variables between groups. If the expected frequency was > 5, Pearson’s chi-square test was adopted for dichotomous variables. If the expected frequency was not > 5, Fisher’s exact test was adopted. *P* < 0.05 was considered statistically significant.

## Results

Twenty-five patients in the observation group and 28 patients in the control group completed the follow-up, with an average follow-up of 18.4 ± 5.2 months (range 13–34 months). Five patients were unable to be followed because of migration and contact information changes (3 patient in the observation group and 2 patients in the control group). All operations were performed by the same surgeon.

General data for the two groups are summarized in Table 1. There was no significant difference in sex, age, BMI, follow-up time, causes of injury, time from injury to surgery, side of knee injury, side of meniscus injury, tear zone of the meniscus, tear length of the meniscus, or number of meniscal repair systems used (Table 1). However, the surgery time in the observation group (85.5 ± 10.6 min) was significantly longer than that in the control group (64.8 ± 11.5 min, t = 3.78, *P* < 0.05).

**Table 1:** General data for the observation group and control group

General data	Observation group (*n* = 25)	Control group (*n* = 28)	*P*
Sex (Male/Female)	16/9	18/10	0.327
Age (year)	25.5 ± 2.3	26.3 ± 1.9	0.513
BMI (kg/m^2^)	23.1 ± 1.3	22.8 ± 1.2	0.139
Follow-up time (month)	18.1 ± 4.7	18.5 ± 5.1	0.402
Causes of injury (sport injury/non-sport-related injury)	20/5	24/4	0.248
Time from injury to surgery (day)	35.5 ± 10.6	38.3 ± 11.4	0.185
Side of knee injury (right/left)	13/12	15/13	0.356
Side of meniscus injury (lateral/medial)	7/18	8/20	0.704
Tear zone of the meniscus (red/red-white)	23/2	25/3	0.633
Tear length of the meniscus (cm)	1.8 ± 0.6	1.6 ± 0.8	0.541
Number of meniscal repair systems used	2.1 ± 0.2	2.2 ± 0.3	0.854
Surgery time (min)	85.5 ± 10.6	64.8 ± 11.5	< 0.05

At the follow-up 1 year after the operation, the knee ROM of the two groups was unlimited, with an average ROM of 125.2 ± 4.3 degrees in the observation group and 124.7 ± 3.8 degrees in the control group. The postoperative pivot shift test for the two groups were all negative. The Lachman test was grade I in 8 patients in the observation group and in 10 patients in the control group, and the other tests were negative.

According to the standard of Barret, 1 patient had knee joint space tenderness and a positive McMurray sign, and 1 patient had slight swelling of the knee joint after activity without obvious pain in observation group; 2 patients had slight swelling of the knee joint after activity without obvious pain in the control group, and the others had no positive symptoms. The clinical healing rate of the meniscus was 92% (23/25) in the observation group and 92.9% (26/28) in the control group. According to the postoperative MRI, the healing rate of the menniscus was 72% (18/25) in the observation group and 71.4% (20/28) in the control group; 2 patients had clinical symptoms associated with the meniscus in two groups respectively and the others had no relevant clinical symptoms.

At the follow-up 1 year after the operation, the IKDC, Lysholm and Tegner scores for the observation and control groups were 90.52 ± 2.8, 89.17 ± 3.1, and 6.81 ± 1.7 and 91.42 ± 1.9, 90.32 ± 3.4, and 7.02 ± 1.4 respectively, and the differences were not statistically significant (*P* > 0.05). A comparison of all the postoperative follow-up data between the two groups is shown in Table 2.

**Table 2:** Comparison of postoperative follow-up data between the two groups

Follow-up data	Observation group (*n* = 25)	Control group (*n* = 28)	*P*
Knee ROM	125.2 ± 4.3	124.7 ± 3.8	0.742
Lachman test (grade I/negative)	8/17	10/18	0.433
Clinical healing rate of meniscus (%)	92% (23/25)	92.9% (26/28)	0.521
MRI healing rate of menniscus (%)	72% (18/25)	71.4% (20/28)	0.292
IKDC score	90.52 ± 2.8	91.42 ± 1.9	0.095
Lysholm score	89.17 ± 3.1	90.32 ± 3.4	0.357
Tegner score	6.81 ± 1.7	7.02 ± 1.4	0.208

## Discussion

The purpose of this study was to explore the practicability and effectiveness of the modified cross-suture method for arthroscopic remediation of unilateral suture anchor pulling out of an all-inside meniscal repair system. The short-term follow-up result of this study show that the modified cross-suture method is practicable and effective for arthroscopic remediation of unilateral suture anchor pulling out during all-inside meniscal repair.

With increased understanding of meniscus structure and function, the treatment concept for meniscus injuries has changed from “If it is torn, take it out!” to “Save the meniscus!” [6, 17]. Increasingly, studies have suggested [18–20] that meniscus injury or defects lead to an increase in the incidence of osteoarthritis; thus, meniscal repair has received increasing attention. Among the repair methods for meniscus injury [21], meniscal suture repair is the mainstream repair method at present and includes three types of repair: outside-in repair, inside-out repair and all-inside repair. Although the technique of inside-out repair is the gold standard for the treatment of body and posterior angle meniscal tear, clinical studies have shown [22–25] that the clinical efficacy of all-inside repair is the same as that of inside-out repair, and that all-inside repair has a shorter operation time and fewer complications. Therefore, all-inside repair for meniscal tears of the body or posterior angle has seen increasing use [26–28].

All-inside meniscal suture repair technology was first reported by Professor Craig Morgan in 1991 [29]; however, this technology still requires an additional incision, and the operation is relatively complex. With the development of science and technology, a new generation of all-inside meniscal repair system, including the OMNISPAN™ meniscus repair system, has seen widespread use [30, 31]. The OMNISPAN™ meniscus repair system consists of OMNISPAN anchor implants and needles, a sterile and disposable deployment gun, a malleable graft retractor, and an arthroscopic pusher/cutter. The OMNISPAN anchor implant is a combination of two molded polyetheretherketone (PEEK) implants combined with 2/0 ORTHOCORD® violet braided composite sutures. The deployment gun properly introduces the anchor implant into the meniscus, and the pusher/cutter facilitates the final suture position to be flush with the meniscal surface. The molded anchor implants along with the suture provide compression across the tear in the meniscus to close the meniscal tear tightly and promote meniscal healing. Biomechanical studies have shown [32] that all-inside meniscal repair and inside-out meniscal repair can achieve similar biomechanical properties after up to 10,000 cycles of loading.

With the increasing popularity of the all-inside meniscal suture repair system, a growing number of doctors have begun to use this type of system to repair the meniscus; however, it requires arthroscopic operation skill and has a relatively steep learning curve, and thus operational errors may occur during meniscal suture repair if there is inadequate surgical experience, inadequate operation visibility, or unskilledl operation assistants. When the deployment gun is misemployed or a needle is hindered by the femoral condyle, the two anchor implants may be introduced into the meniscus simultaneously, or one anchor implant may fall off or be pulled out. In these cases, only the unilateral anchor play the role of fixation and the repair system fails, especially in the repair of the medial meniscus [11–13]. Under these circumstances, the meniscal repair system is usually completely removed, and a new repair system is implanted [11].

In clinical practice, some oblique or cross-suture methods have been applied in the treatment of radial tears of the meniscus and have achieved good results [33–35]. Biomechanical studies have shown [36] that oblique sutures have the dual advantage of vertical sutures (superior biomechanical strength) and horizontal sutures (ease of application, longer sutures with a tendency to cover a larger meniscal tissue area). According to such theoretical and practical results, we hypothesize that if these methods are applied to remediate the unilateral suture anchor pulling out of an all-inside meniscal repair system, the clinical efficacy of this method may be improved. Using the shoulder arthroscopic knotting technique, the suture from the unilateral anchor pulling out and the other suture from the normal side were cross-knotted to form an oblique suture, where the optimal fixation effect could be achieved by tension adjustment of the knot. We named this the “modified cross-suture” method. At present, we are actively exploring this system. After our short-term follow-up, we found that this method was practicable and effective, and was suitable for the remediation of one or more unilateral anchors pulling out. Compared with the control group, the observation group had a longer operative time. However, the clinical efficacy of the two groups was similar, and was also comparable to that reported for other conventional suturing methods [37, 38]. Therefore, when the meniscus tear length is long and unilateral pulled-out anchor happened during all-inside meniscal repair, this method can reduce the number of new implanted meniscus repair systems needed.

To achieve better clinical efficacy when exploring new surgical methods, all patients in this study underwent ACL reconstruction concurrently to improve the healing rate of the meniscal suture repair. At present, many studies have shown [39–41] that concurrent ACL reconstruction has little influence on the healing rate of meniscal suture repair and that the injured area of the meniscus is a key factor affecting the healing rate. Based on the results of this study, if the clinical efficacy of our method is confirmed in the long-term follow-up, this method can be applied to more patients to improve clinical efficacy and medical economic benefits.

### Limitations

There are several limitations in this study. First, the relatively small number of cases limits the overall validity of our findings. However, modified cross-suture repair is relatively rare and only cases with unilateral suture anchor pulling out were included. Therefore, our study cohort represents a relatively homogenous group in terms of surgical treatment. Second, the follow-up time was relatively short. Therefore, longer follow-up is needed to determine the long-term effectiveness of the proposed method. Third, further prospective multicentre studies and biomechanical studies are necessary to evaluate the practicability and effectiveness of the modified cross-suture technique for meniscal repair. Fourth, to place many anchors of the modified cross-suture method may increase the risk of meniscal cyst. Although meniscal cyst have not been found in this study, it still needs long-time observation.

## Conclusion

The modified cross-suture method is practicable and effective for arthroscopic remediation of unilateral suture anchor pulling out in an all-inside meniscal repair system.

## Data Availability

The datasets used and analysed during the current study are available from the corresponding author on reasonable request.

## Abbreviations

ACL: Anterior cruciate ligament
MRI: Magnetic resonance imaging
IKDC: International Knee Documentation Committee
ROM: Range of motion
